# Phlegmasia cerulea dolens: a rare cause of shock

**DOI:** 10.1002/rcr2.424

**Published:** 2019-04-08

**Authors:** Christopher Bob Lewis, Matthew Kevin Hensley, Julie Elizabeth Barrett, Steven Burke Van Norman, Alexander Stuart Taylor, Jeffrey Craig Horowitz

**Affiliations:** ^1^ Department of Internal Medicine University of Michigan Ann Arbor Michigan USA; ^2^ Department of Internal Medicine, Division of Pulmonary and Critical Care University of Michigan Ann Arbor Michigan USA; ^3^ Department of Internal Medicine and Pediatrics University of Michigan Ann Arbor Michigan USA; ^4^ Department of Pathology and Clinical Laboratories University of Michigan Ann Arbor Michigan USA

**Keywords:** Inferior vena cava filter, multiple organ failure, vascular diseases, venous thromboembolism

## Abstract

Phelgmasia cerulea dolens (PCD) is a rare cause of shock that can complicate deep venous thrombosis and carries a high risk of mortality. We present a case of extensive bilateral lower extremity deep vein thrombosis associated with an inferior vena cava filter which rapidly progressed to PCD and refractory shock.

## Introduction

Phlegmasia cerulea dolens (PCD) is a complication of acute deep venous thrombosis (DVT) characterized by pain, swelling, and skin discolouration. Venous obstruction can lead to compartment syndrome, venous gangrene, and death in 25%–40% of cases 1. Common risk factors for PCD include malignancy and hypercoagulable states, and prompt intervention is necessary to prevent catastrophic complications. We present a case of PCD and shock associated with thrombosis of a permanent inferior vena cava (IVC) filter.

## Case Report

A 51‐year‐old male with a permanent IVC filter that had been inserted approximately 20 years ago when the patient developed a DVT during a hospitalization for severe non‐ischaemic cardiomyopathy, was transferred to our medical intensive care unit for shock and acute renal failure. Following the IVC insertion, he had been treated with warfarin for one year and had been on anti‐platelet therapy since.

He had been admitted to the hospital three days prior to transfer after presenting with progressive bilateral lower extremity pain and decreased sensation in his gluteal region. Acute bilateral DVTs involving the common femoral and popliteal veins were diagnosed. Over 48 h, despite receiving unfractionated heparin, he developed anuric renal failure and shock. Placement of a right internal jugular dialysis catheter was complicated by airway compromise due to a retropharyngeal haematoma necessitating endotracheal intubation. The heparin infusion was discontinued and the patient was transferred to our hospital.

**Figure 1:**
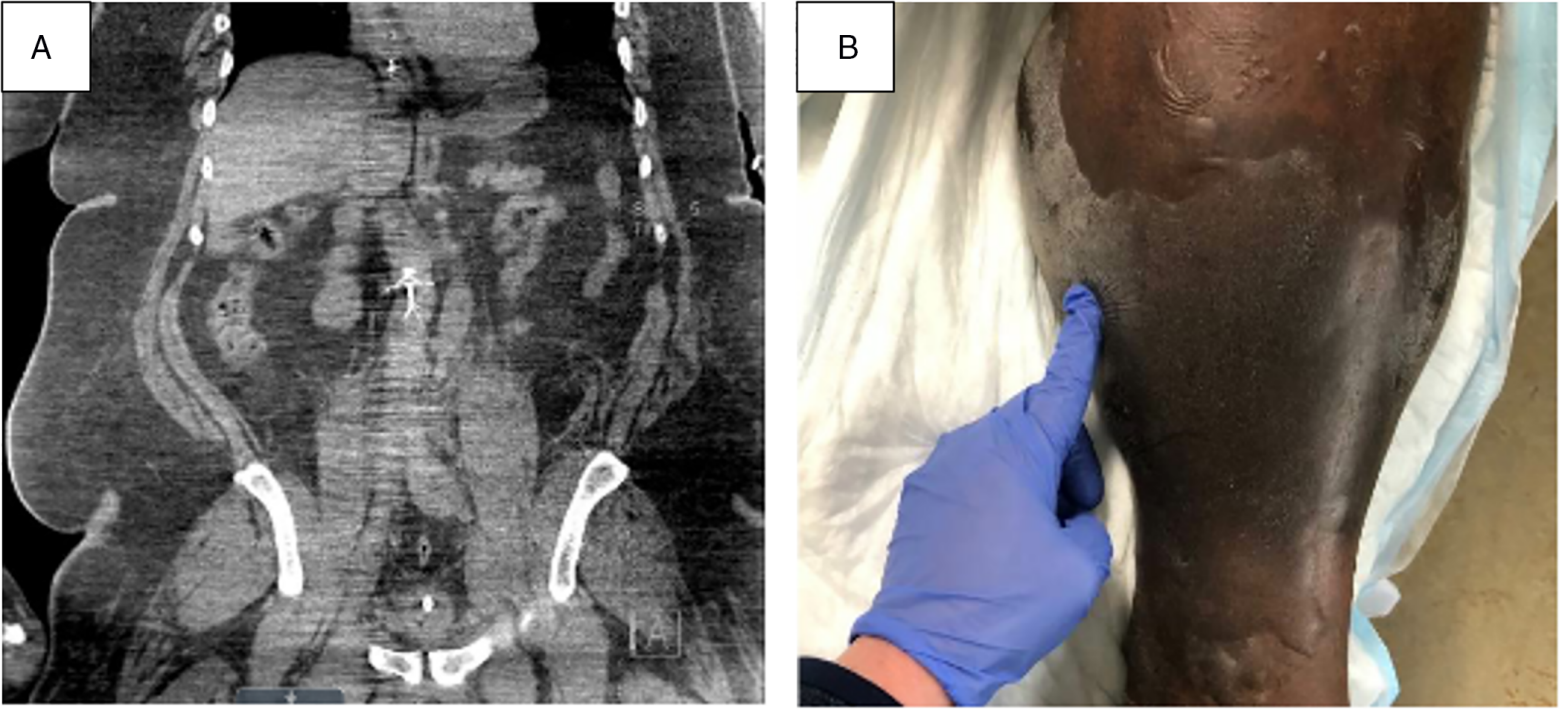
(A) Coronal view of the computed tomography imaging of the abdomen and pelvis shows dilation of the distal inferior vena cava below the level of the inferior vena cava filter. (B) Tense lower extremity oedema with discolouration and fluid‐filled bullae.

On arrival, his mean arterial pressure was 71 (104/53) mmHg while on norepinephrine, vasopressin, and phenylephrine. Arterial blood gas analysis showed a pH of 7.06, partial pressure of carbon dioxide (PaCO_2_) of 28 mmHg, partial pressure of oxygen (PaO2) of 312 mmHg, and a lactate of 16 mmol/L. The platelet count was 31 K/μL. Examination was notable for tense bilateral lower extremity oedema. Dorsalis pedis pulses were detectable with Doppler ultrasound. An abdominal computed tomography (CT) showed dilation of the distal IVC suggesting thrombosis (Fig. 1A). Transthoracic echocardiography showed a 25% ejection fraction with no right ventricular dilation or strain. The IVC was collapsible proximal to the hepatic veins. Lower extremity ultrasound confirmed acute bilateral DVTs involving the external iliac and femoral veins. Laboratory evaluations excluded thrombophilia, heparin‐induced thrombocytopenia and thrombotic thrombocytopenic purpura. Infusion of 5 L of isotonic fluid and continuous renal replacement therapy led to a reduction in the vasopressor requirement, a reduction in lactate to 2.0 mmol/L, and pH/PaCO_2_ normalization. However, the lower extremity oedema progressed with development of bullae and purple skin discolouration (Fig. 1B). Dorsalis pedis pulses became undetectable, consistent with compartment syndrome due to PCD.

**Figure 2:**
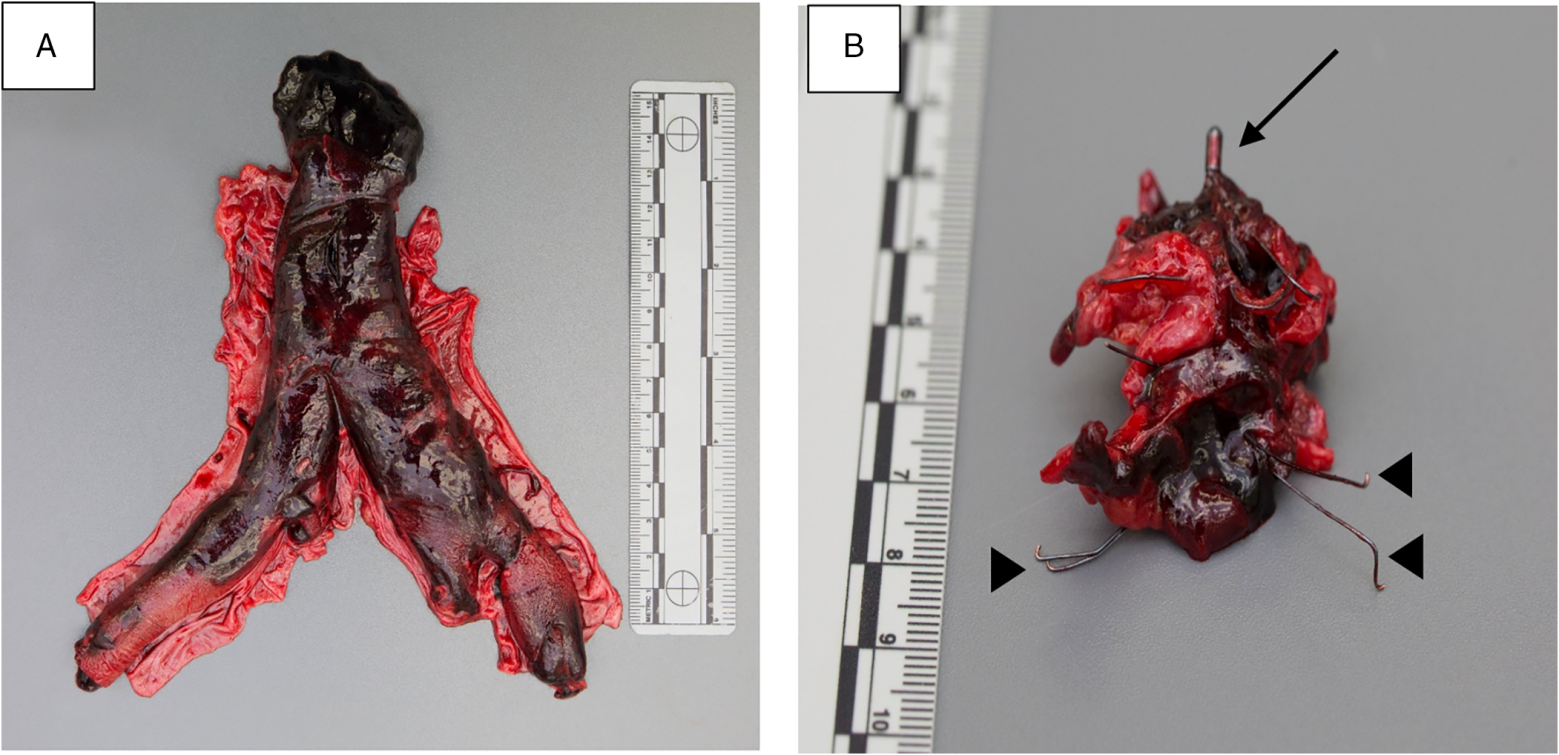
(A) Extensive thrombosis in the inferior vena cava (IVC) extending into the bilateral iliac and femoral veins. (B**)** Proximal extent of the IVC thrombus surrounds the IVC filter, which can be seen extruding from the thrombus.

Catheter‐directed thrombolysis, surgical thrombectomy, and fasciotomy were deemed to be contraindicated due to ongoing shock, severe cardiomyopathy, the retropharyngeal haematoma, and persistent thrombocytopenia thought to be the consequence of platelet consumption. Unfractionated heparin was restarted and, within 24 h, lower extremity pulses were again palpable. However, there was a progressive rise in creatinine phosphokinase to 44,000 IU/L and an increase in lactate to 5.8 mmol/L despite continued vasopressor support and continuous dialysis. His family decided to pursue palliation and withdrawal of life‐supportive measures. Post‐mortem examination confirmed an occluding thrombus at the level of the IVC filter with extension to the internal and external iliac veins (Fig 2A, B). The autopsy did not identify an underlying malignancy.

## Discussion

Early intervention for PCD with anticoagulation, thrombolysis, or thrombectomy is essential, as progression to venous gangrene portends mortality in up to 57% of cases 1. In our case, bilateral venous gangrene and compartment syndrome developed and progressed rapidly following cessation of anticoagulation in the setting of a retropharyngeal haematoma.

The introduction of IVC filters in 1967 was followed by a progressive increase in their use 2. Thrombosis of the IVC has been reported in up to 22% of patients with IVC filters in place for a median duration of eight months 3, although anticoagulation diminishes the likelihood of this complication 4. The clinical presentation of IVC filter thrombosis depends on the acuity of occlusion, as the lack of collateral vessels in the setting of an acute thrombus can impede venous return. Although rare, IVC filter thrombosis with PCD leading to compartment syndrome and death without surgical intervention has been reported 4. The prolonged duration between IVC filter placement and the development of an associated acute obstructing IVC thrombus in our patient is atypical, but demonstrates that the risk of such events can persist over time.

Complications associated with permanent IVC filters, including IVC thrombosis, spurred the development of retrievable IVC filters in the 1990s but these devices were not approved in the United States until 2004 5. Thus, the IVC filter in our patient was unlikely to have been designed for retrieval. Interestingly, a recent study found that despite a 200% increase the use of these “temporary” IVC filters, the actual retrieval rates remain quite low (12%–45%) 5.

Beyond malignancy and thrombophilia, the risk factors for IVC filter thrombosis remain poorly defined. An association with cardiomyopathy has not been reported 3. We speculate that in our patient the cardiomyopathy contributed to clot formation and accelerated the progression to compartment syndrome. Thus, while demonstrating the critical role of early recognition and intervention in cases of PCD, this case underscores the long‐term risks associated with IVC filters and highlights the importance of IVC filter removal whenever it is feasible. Finally, this case illustrates the importance of starting anticoagulation as soon as possible and suggests that anticoagulation be maintained indefinitely in patients with permanent IVC filters.

### Disclosure Statement

Appropriate written informed consent was obtained for publication of this case report and accompanying images.
